# Intestinal Microbiota in Healthy Adults: Temporal Analysis Reveals Individual and Common Core and Relation to Intestinal Symptoms

**DOI:** 10.1371/journal.pone.0023035

**Published:** 2011-07-28

**Authors:** Jonna Jalanka-Tuovinen, Anne Salonen, Janne Nikkilä, Outi Immonen, Riina Kekkonen, Leo Lahti, Airi Palva, Willem M. de Vos

**Affiliations:** 1 Department of Veterinary Biosciences, University of Helsinki, Helsinki, Finland; 2 Research & Development, Valio Ltd, Helsinki, Finland; 3 Laboratory of Microbiology, Wageningen University, Wageningen, The Netherlands; Charité-University Medicine Berlin, Germany

## Abstract

**Background:**

While our knowledge of the intestinal microbiota during disease is accumulating, basic information of the microbiota in healthy subjects is still scarce. The aim of this study was to characterize the intestinal microbiota of healthy adults and specifically address its temporal stability, core microbiota and relation with intestinal symptoms. We carried out a longitudinal study by following a set of 15 healthy Finnish subjects for seven weeks and regularly assessed their intestinal bacteria and archaea with the Human Intestinal Tract (HIT)Chip, a phylogenetic microarray, in conjunction with qPCR analyses. The health perception and occurrence of intestinal symptoms was recorded by questionnaire at each sampling point.

**Principal Findings:**

A high overall temporal stability of the microbiota was observed. Five subjects showed transient microbiota destabilization, which correlated not only with the intake of antibiotics but also with overseas travelling and temporary illness, expanding the hitherto known factors affecting the intestinal microbiota. We identified significant correlations between the microbiota and common intestinal symptoms, including abdominal pain and bloating. The most striking finding was the inverse correlation between Bifidobacteria and abdominal pain: subjects who experienced pain had over five-fold less Bifidobacteria compared to those without pain. Finally, a novel computational approach was used to define the common core microbiota, highlighting the role of the analysis depth in finding the phylogenetic core and estimating its size. The in-depth analysis suggested that we share a substantial number of our intestinal phylotypes but as they represent highly variable proportions of the total community, many of them often remain undetected.

**Conclusions/Significance:**

A global and high-resolution microbiota analysis was carried out to determine the temporal stability, the associations with intestinal symptoms, and the individual and common core microbiota in healthy adults. The findings provide new approaches to define intestinal health and to further characterize the microbial communities inhabiting the human gut.

## Introduction

Following birth, our gastrointestinal (GI) tract is colonized by a myriad of microbes, collectively termed the (GI) microbiota, that develop intimate interactions with our body and contribute to our health and well-being [1]. Altered GI microbiota has been detected not only in intestinal aberrations, such as inflammatory bowel diseases [2] and the milder irritable bowel syndrome (IBS) [3], [4] but also in systemic diseases including type 2 diabetes and other metabolic diseases [5], [6]. Due to the health relevance of the GI microbiota, the characterization of its diversity and function is actively ongoing with recently developed molecular high-throughput technologies, such as next generation technology sequencing of 16S rRNA amplicons and phylogenetic microarrays [7], [8].

Several studies have attempted to describe the normal GI microbiota in healthy individuals but due to the strong individual variation, low-resolution measurement methodologies and limited number of samples the task is challenging. Host-specificity and relative temporal stability of the GI microbiota has been well established using molecular methods [9]–[14]. The temporal stability of the overall microbiota suggests the existence of an individual core, consisting of microbes that retain within an individual over time [7]. The temporal stability may also reflect the resilience of the ecosystem; even strong perturbations such as antibiotics have mainly short-term effects on the dominant microbiota [15]–[17]. There is evidence for a negative correlation between the time span and the within-individual microbiota similarity already in months scale [13], [18] possibly reflecting the cumulative effect of environmental perturbations.

The occurrence of intestinal symptoms and their possible effect on the quality of life in healthy individuals is largely unknown. In an American survey, almost half of the general population reported one or more intestinal symptoms within one month, the most common complaints being diarrhea or loose stools, abdominal pain or discomfort, and bloating or distension [19]. The high prevalence of intestinal problems in healthy subjects suggests their substantial impact on the general well-being. Accordingly, IBS patients, suffering from various intestinal symptoms, have significantly impaired quality of life in comparison to the general population [20], [21]. The Health-Related Quality of Life (HRQoL) can be measured with validated questionnaires that evaluate the subject's general, psychological and social well-being [22]. It is of particular relevance for the emerging interdisciplinary field of gut health research [23] to find out to which extent the intestinal symptoms of healthy individuals impair their quality of life and whether the occurrence of intestinal symptoms is associated to the GI microbiota composition.

A fundamental question pertaining to the GI microbiota characterization is the existence of a common core microbiota that we all may share. The so far presented studies of this common core GI microbiota are based on the analysis of 16S rRNA gene sequence inventories [12], [14], [24]–[27] or phylogenetic microarray analysis [13]. Most of the studies indicate only a small phylogenetic overlap between the individuals. The gene pool of the GI microbiota, in turn, appears conserved across the individuals suggesting functional redundancy of the ecosystem [8], [12]. However, the variable abundance between species has so far been ignored, despite its crucial impact on whether a particular species is detectable in a given analysis depth and consequently, on the extent of shared bacteria. The increase in sequencing depth has been shown to markedly increase the core size as it resulted in the coverage of those phylotypes that had low abundance in some of the individuals [8]. It is difficult to the compare the existing common core studies because of the number of the study subjects varies, their health status has not been determined and the required prevalence for the core species has not been defined [8], [12], [24] **.** Moreover, the results are affected by methodological aspects including the DNA extraction methods [28], [29], the choice of the hypervariable region of the 16S rRNA [12], the coverage of the selected primers, the analysis depth [8], [25], the phylogenetic assignment and cut off values [30], and the selected bioinformatics pipeline [31]. As a consequence of the variable study set-ups, there is currently no consensus about the size and composition of the common core GI microbiota.

The present study expands the present concept of temporal dynamics of the overall microbiota and its individual members, based on frequent sampling and a comprehensive microbiota analysis with a phylogenetic microarray. Furthermore, we analyze for the first time, to our knowledge, the associations between the intestinal microbiota and intestinal symptoms in healthy adults. We observed that the abundance of several taxa is associated with intestinal symptoms including abdominal pain and bloating. We also add a novel perspective to the characterization of the common core microbiota. We used a sequencing-independent technology and a novel computational approach, which is not restricted to prefixed criteria for the phylotype abundance or prevalence but instead studies how these analytical parameters impact the resulting core size. Our results highlight the influence of analysis depth and suggest that when encompassing also the minor microbiota, a substantial proportion of the intestinal phylotypes appear to be shared between the individuals.

## Results

### Definition of the health status

To monitor the subjects' health, we asked the participants to record their general, intestinal, and emotional health status at each fecal sampling point. To focus on the healthy, uncompromised subjects, and to exclude the potentially confounding samples from further analyses, we calculated the HRQoL scores and utilized them in parallel to the background information to categorize the participants (Table 1). Of the 15 participants, nine subjects qualified as healthy based on their high general health score that was comparable to the Finnish reference values [32], high (>90) GI health score, and the absence of any adverse events during the study period. The remaining subjects were considered as compromised: Subject 9 received antibiotic treatment, and subjects 2 and 4 suffered from considerable intestinal symptoms (bloating, constipation and abdominal pain) that decreased their GI health scores significantly compared to those of the healthy (t-test p-value <0.05) and negatively affected their general health perception (Table 1). The remaining three subjects were classified as compromised based on the combination of exploratory HITChip data analysis and background information (see next section).

**Table 1:** Demographic table, assigned health status (see text for details) and the HRQoL scores of the study subjects.

Subject ID	Gender	Age (SD)	BMI (SD)	Assigned health status Healthy (H) or Compromised (C)	General health score* (SD)	GI symptom score* (SD)	Mental health score* (SD)
1	m	54	26.8	H	75 (0)	100.0 (0.0)	80.8 (0.0)
2	f	53	20.3	C (GI symptoms)	66.7 (5.3)	68 (21.8)	58.6 (7.4)
3	f	51	31.7	H	58.3 (5.3)	95.7 (3.3)	76.3 (5.1)
4	m	44	20.7	C (GI symptoms)	47.2 (28.7)	63.3 (27.2)	52.6 (20.2)
5	f	42	21.5	H	79.2 (4.6)	98.3 (2.0)	93.9 (5.3)
6	f	56	29.1	C (Traveler)	73.6 (6.3)	99.8 (0.6)	75.0 (3.2)
7	f	30	17.6	H	70.8 (14.7)	92.9 (4.0)	76.9 (4.4)
8	m	30	23	C (Traveler)	79.2 (11.5)	96.1 (2.9)	80.8 (5.0)
9	f	40	23.6	C (Antibiotics)	55.6 (22.8)	94.1 (1.3)	65.4 (8.9)
10	f	27	20.8	H	79.2 (14.7)	92.9 (3.7)	85.6 (2.7)
11	f	54	26.1	C (Traveler)	68.1 (16.2)	90.7 (3.2)	64.7 (10.5)
12	m	51	25.9	H	68.1 (8.2)	96.6 (1.2)	69.2 (13.5)
13	m	47	24	H	65.3 (3.4)	96.1 (0.8)	78.5 (1.4)
14	f	45	21.7	H	83.3 (5.3)	98.8 (1.1)	85.9 (4.0)
15	f	39	21.3	H	68.1 (3.4)	99.0 (1.8)	80.4 (3.3)
**Average**	**5 m/10 f**	**44.2 (9.5)**	**23.6 (3.3)**	H	**69.2 (9.9)**	**92.1 (11.1)**	**75.0 (11.0)**

*The health scores represent average from the six time points.

### Comparative analysis of the subjects' intestinal microbiota over time

**Figure 1:**
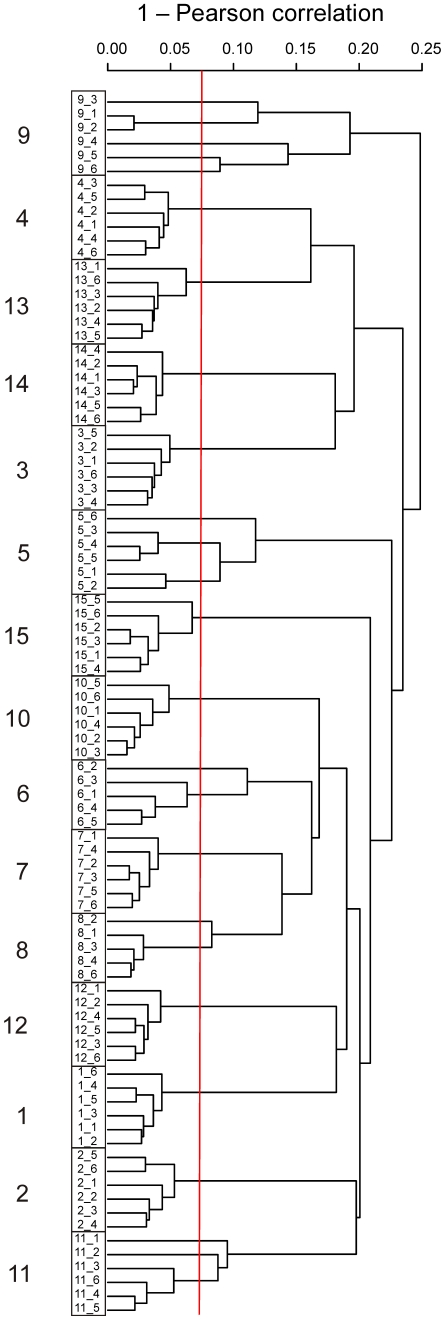
Hierarchical clustering of the HITChip profiles of 15 subjects and their six timepoints. The subject-wise clustering is highlighted with boxes and the temporal variation is displayed with the variable length of the branches. Red vertical line is drawn at Pearson correlation of 0.925, below which the intra-individual sample similarity was detected only in the five unstable subjects (5,6,8,9 and 11) discussed in the text.

To obtain an overview of the similarity of the total GI microbiota between all the study subjects, we unsupervisedly grouped the preprocessed HITChip profiles (see Methods and Figure S1) using hierarchical clustering with correlation distance method (Figure 1). The largest differences in the microbiota profiles were the inter-individual differences (mean inter-individual Pearson correlation; r = 0.78, standard deviation; ±0.04). All samples clustered in a subject-wise manner (Figure 1), and a high overall stability of the individuals' microbiota was observed (mean intra-individual r = 0.96, ±0.02 over all time points).

Despite the overall stability we detected substantial variation in the temporal dynamics of the subjects' microbiota as visualized by the variable lengths of the branches in the clustering tree. Subject 9 was on an antibiotic course during the collection of the fourth sample, which separated clearly from the other samples (Figure 1). Also the preceding sample taken two weeks earlier branched deeply in the dendrogram implying a distinct microbiota profile. At that time the subject was not medicated but was ill and few days later diagnosed with streptococcal pharyngitis. Apart from the antibiotic treatment, we observed no correlations between the microbiota composition or stability and the recorded medication (subjects 1,3,7 and 11 received regularly thyroid or estrogen hormones, or hypertension treatment). A decreased temporal stability was also observed in subjects 5, 6, 8 and 11 (Figure 1). For all these subjects, with the exception of subject 5, the decreased stability could be explained in the light of the background information. Subjects 6, 8 and 11 had records of travelling during the first weeks of the trial, passing different time zones in a range of 1–7 hours. The similarity between the samples taken before and after the travelling was statistically significantly lower than between the samples taken two to four weeks after travelling (r = 0.90, ±0.02 and 0.96, ±0.02, respectively; t-test p-value <0.05) (Figure S2). Accordingly, the travelers showed significantly lower intra-individual stability of the GI microbiota than the nine healthy subjects (r = 0.93±0.01 and 0.96, ±0.02, respectively; t-test p-value <0.05), suggesting that the GI microbiota was affected by the travel. Therefore, although the travelers were healthy, we classified them in this context as compromised together with the three subjects affected by an antibiotic treatment or persistent intestinal symptoms (Table 1). The rationale for such classification was to focus the compositional microbiota analysis on those healthy subjects for which we did not observe any traceable destabilizers.

### Compositional microbiota analysis

**Figure 2:**
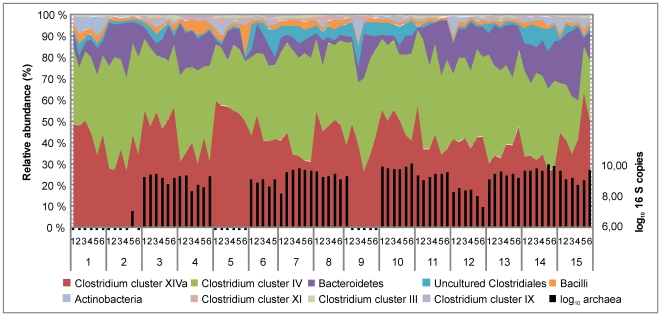
Microbiota composition of the study subjects. Relative abundance of the phylotypes that contributed over 0.5% to the total HITChip signal was summed up to phylum level, except Firmicutes, which were summed up to class or Clostridium cluster level (left y-axis). The qPCR-based quantification of the methanogens is shown on the right y-axis.

The GI microbiota in the nine healthy uncompromised subjects was analyzed in detail using HITChip signal intensities of species-like taxa that we hereafter term phylotypes (level 3 phylogeny in Methods and [13]). We detected on average 470 phylotypes per subject, representing 68.4% of the total number of the phylotypes that passed the signal treshold (Figure S1). The most abundant phylum in the study subjects consisted of Firmicutes with over 80% contribution to the total microbiota, followed by an average 10% of Bacteroidetes and 1.5% of Actinobacteria (Figure 2). Verrucomicrobia and Proteobacteria were detected with proportional share below 1% (data not shown). As expected, the most abundant Firmicutes were the members of Clostridium clusters XIVa and Clostridium cluster IV, comprising of an average proportion of 40% and 35% of the total microbiota, respectively.

Based on *Methanobrevibacter*–specific quantitative PCR (qPCR), intestinal methanogens were present in 80% of the individuals (12/15). In one subject (subject 2; Figure 2) the methanogens were detected only in a single timepoint, while other 11 carriers had them in all the timepoints. The abundance of the methanogens varied over two orders of magnitude between the carriers, ranging from log10 counts of 7.1 to 10.2 g-1 feces. The relative proportion of the methanogenic archaea in the total GI microbiota was determined by the ratio of the 16S rRNA gene copy numbers of the methanogens and the total bacteria. The proportion of the methanogens was below 1% in most of the samples while in subject 14 the methanogens represented 1–5% of the total community across the timepoints.

### Temporal variation of the healthy microbiota and the individual core

We analyzed the effect of time on the microbiota variation in the nine healthy subjects by determining the similarity of the HITChip profiles between the intra-individual samples. The similarities were determined by a correlation coefficient computed for all possible time intervals from one to seven weeks. The mean intra-individual Pearson correlations ranged from 0.96 (±0.02; one week interval) to 0.95, (±0.02; seven week interval), showing only a slight, non-significant, decline in the microbiota similarity over time. In order to address the individual core consisting of microbes that retain over time, we determined the taxon-specific temporal variability by calculating the Coefficient of Variation (CoV) within a subject for each phylotype. The CoVs ranged from 0.83% (uncultured *Ruminococcus callidus*-like bacterium) to 46.1% (phylotype within uncultured Clostridiales) with an average of 6.3% (±1.1%), reinforcing the high overall stability. Among the 50 most stable genus-level taxa per subject, the most common statistically enriched genus-level groups were *Ruminococcus obeum* and relatives (*et rel.*), affiliated to the recently renamed genus *Blautia* [33] with an average CoV of 3.7%, and *Clostridium symbiosum et rel.* with an average CoV of 4.4% (Table S1). No other stable genus-level taxon was shared by >50% of the healthy subjects, indicating subject-specificity of the stable species, i.e. of the individual core microbiota. Similarly, we determined the 50 statistically significant, temporally most unstable phylotypes per each healthy subject (Table S2). The most common unstable genus-like taxa were uncultured Clostridiales and *Bacteroides vulgatus et rel.* (CoV 9.9%), but even they were common to only few individuals. Not only the commonness but also the actual CoVs of the unstable groups had larger inter-individual variation than the stable ones, indicating that no genus-level group was generically unstable. Few genus-like groups, including *F. prausnitzii et rel.*, were in the top list of both stable and unstable taxa (Tables S1 and S2), highlighting the large individual variation in the temporal behavior of these taxa. In contrast, the methanogenic archaea appeared temporally very stabile among the carriers, as the intra-individual CoVs calculated from the qPCR data were limited to a range of 1% to 6%.

### Correlation between intestinal symptoms and the GI microbiota

**Figure 3:**
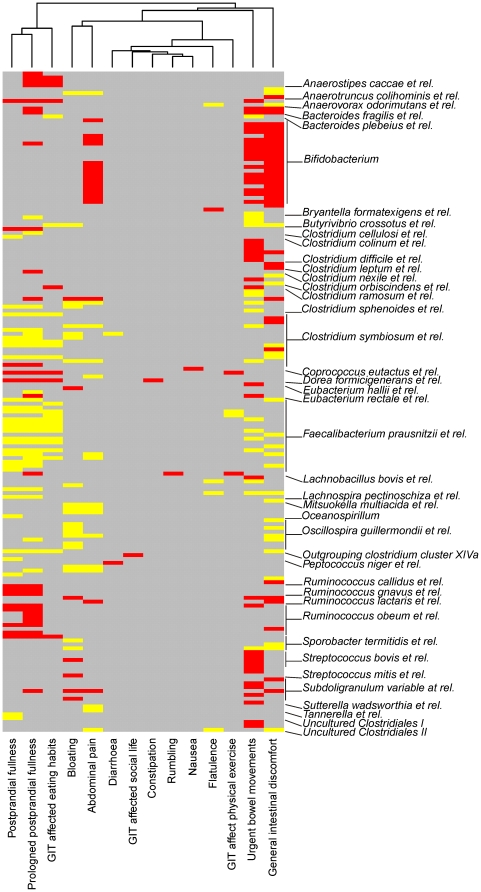
Correlation heatmap of the microbiota profiles and intestinal symptoms. The heatmap visualizes all significant (q-value <0.005) Spearman correlations between the intestinal symptoms and the HITChip-measured abundance of bacterial phylotypes. Each row represents a single phylotype, whose genus-level assignments are listed below the rows, or in the middle when several rows refer to the same genus-level group. The negative correlations between symptoms are indicated in red and the positive correlations with yellow; non-significant correlations are shown in gray.

Despite the good general health of the participants, only one out of the 15 subjects did not experience any intestinal complaints during the study period (subject 1; Table 1). However, occasional and mild intestinal problems dominated the answers as only two subjects had intestinal health score below 90 out of 100 (Table 1). To detect the bacteria associated to specific intestinal symptoms, we correlated the HRQoL–questionnaire scores and the microbiota using the HITChip data from all the 15 subjects. We could identify 170 phylotypes, which correlated statistically significantly (q<0.005) with 14 HRQoL-questions related to intestinal symptoms (Figure 3). The significant correlations were most frequently found for general intestinal discomfort, urgent need for defecation, postprandial fullness, and abdominal pain. About half (53%) of the 170 phylotypes correlated negatively with the symptoms, showing higher abundance in the absence of the symptoms. The negatively correlating genus-like taxa included Bifidobacteria, *R. obeum et rel.*, *Streptococcus bovis et rel.*, *Clostridium difficile et rel.* (mostly due to the nonpathogenic *Clostridium irregularis)* and Ruminococci. The other half showed a positive correlation with the observed symptoms so that the probe intensities were higher when the symptoms occurred. The positively correlating phylotypes represented mainly members of the Clostridium clusters IV and XIVa, among which uncultured members of the *F. prausnitzii et rel.* and *C. symbiosum et rel.* were the most predominant groups.

We next aimed to identify the bacteria related to the symptoms that were most frequently experienced in our survey, namely bloating (31 complaints from 8 subjects) and abdominal pain (28 complaints from 8 subjects). For bloating, the severity of the recorded complaints varied from strong discomfort (2 records) to mild symptoms (23 records). Several uncultured phylotypes from Clostridium clusters IV and XIVa had statistically significant positive correlation with bloating (Table S3). The abundance of phylotypes within *Anaerotruncus colihominis et rel., Ruminococcus callidus* and *Lachnospira pectinoschiza et rel.* were over ten-fold higher when bloating was recorded.

**Figure 4:**
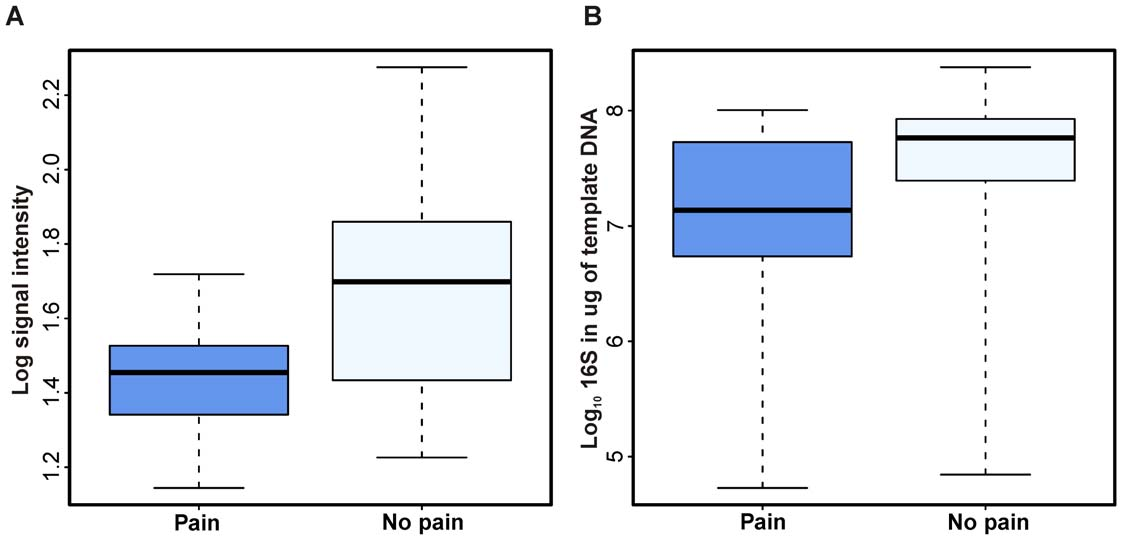
Quantification of Bidifobacteria in relation to abdominal pain. The samples were divided into two groups: with and without concurrent abdominal pain, and the abundance of Bifidobacteria in each group was determined using **A.** HITChip and **B.** qPCR. The difference between the groups was statistically significant (q≤0.02 in A; p<0.05 in B). The box extends from 25th percentile to 75th percentile, with a line at the median; the whiskers extent to the highest and lowest values.

The subjects experienced abdominal pain in varying degree so that the severity of the recorded complaints ranged from very strong disturbance (1 record) to mild symptoms (20 records). The most striking finding related to the abdominal pain was its negative correlation with the abundance of Bifidobacteria (r = −0.45, ±0.03; Table S4 and Figure 3). The majority of the *Bifidobacterium* phylotypes that were above the detection limit (14/21) had statistically significant (q≤0.02) negative correlation with abdominal pain. The subjects who experienced pain had over five-fold (on average 5.4, ±1.3) less Bifidobacteria compared to the subjects without recorded pain (Figure 4A). Moreover, a genus-specific qPCR, covering most cultured members of this group, revealed a statistically significant difference and a fold change of two (Wilcoxon test p<0.05) in the Bifidobacterial counts according to the symptom status (Figure 4B).

In addition to Bifidobacteria, one phylotype within *R. lactaris et rel.*, Clostridium cluster IV, was significantly decreased in pain-associated samples (Table S4). Among the positive correlations, uncultured, potentially pathogenic phylotypes within Uncultured Clostridiales II, *Anaerotruncus colihominis et rel.* and *R. callidus,* were increased over ten-fold when pain was recorded (Table S4). *C. symbiosum* and uncultured phylotypes affiliated to *F. prausnitzii et rel.* were more abundant in subjects experiencing postprandial fullness (Figure 3). The positive correlation between postprandial fullness and the abundance of *F. prausnitzii* was confirmed with qPCR (data not shown). The symptom scores of bloating and abdominal pain correlated strongly (ρ = 0.72). Four genus-level Clostridial groups were significantly correlated with the both symptoms, albeit in a non-uniform direction (Tables S3 and S4).

### The common core microbiota

**Figure 5:**
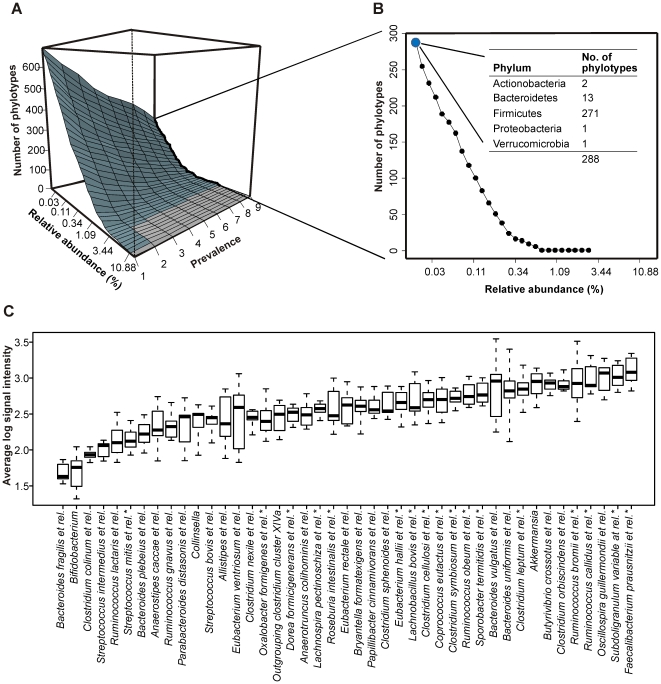
Definition of the common core microbiota. **A.** Perspective plot is used to visualize how the number of phylotypes in the common core is a function of the selected abundance and prevalence. Light gray indicates the area where no phylotypes passed the given criteria. **B. The common core microbiota in the healthy subjects.** The y-axis represents the range of the phylotype count shared, thus forming the common core, in the nine healthy subjects. The line visualizes how the number of the phylotypes depends on the selected percentile abundance, and how it decreases drastically when the phylotypes with lowest abundance are excluded. List shows phylum-level summary of the phylotypes that contributed with over 0.03% relative abundance **C.** **The common core phylotypes summarized to genus-level taxa.** The boxplots visualize the intra-individual variation in the abundances of the core taxa. *Bacteroides fragilis et rel.* and Bifidobacteria (two leftmost boxes) are visualized for reference purposes. These taxa were not part of the common core due to their large intra- and inter-individual variation. For boxplot details, please see the legend for Figure 4.

In-depth phylogenetic microarray analysis provided the opportunity to explore the common core microbiota that is potentially shared between the healthy individuals. Most of the present efforts in determining and defining the microbial core have focused on the presence and absence of phylotypes in a defined proportion of the study subjects. Instead of using arbitrary criteria for the abundance and prevalence of the phylotypes, we addressed the common core microbiota using a novel approach that exploits adjustable values of these two parameters. Considering the relative abundance, we utilized the full dynamic range of the HITChip microarray and incorporated the phylotypes that at lowest comprised as little as 0.02% of the estimated total signal while the most dominant phylotypes had over 5% relative abundance. With regard to the prevalence, we accommodated all possibilities between 0–100% (n = 0–9). The averaged HITChip data from the six timepoints per person were used. As a result, the size of the common core is not a single number but instead a continuum from zero to hundreds, depending on the selected abundance and prevalence values as visualized with a perspective plot (Figure 5A). At the prevalence of 50%, only 10 phylotypes remained in the common core when the minimum abundance was set as high as 0.5% of the estimated total signal. On the other hand, when all the phylotypes above the detection limit were accepted, changing the prevalence from 50% to 100% had much less effect in the number of the core phylotypes. In other words, the number of the shared phylotypes decreased dramatically when a phylotype was required to be in high abundance in all samples, emphasizing the contribution of the low-abundance bacteria in the core.

By including all the phylotypes above the signal treshold in all the nine healthy subjects, we detected 288 phylotypes in the common core (Figure 5B and Table S5). The 288 phylotypes represented 41.9% of all the detected phylotypes, indicating a high degree of phylogenetic overlap between the individuals. The exact taxon numbers should be regarded with caution as they are subject to noise arising e.g. from unintended cross-hybridization with non-target sequences and differences in the probe binding affinities. To estimate such noise, and to exclude the possibility that related probe-level effects would have remarkably affected the common core composition and size, we used the Robust Probabilistic Averaging algorithm designed for probe performance analysis [34]. For the vast majority of the common core phylotypes (234; 81%) the correlation between the probabilistic and the standard method was >0.9, indicating a low proportion of potentially false positives among the detected core phylotypes. The preprocessing did not affect the phylogenetic composition of the core as the 22 phylotypes detected with most unreliable probes (r<0.8) were all assigned to the diverse and polyphylogenic Clostridium clusters XIVa and IV that vastly dominated the core with 145 and 111 phylotypes, respectively (Table S5). The majority of the core organisms were uncultured phylotypes, belonging to 43 different genus-like taxa from five phyla (Figures 5B and 5C). The most represented groups were *R. obeum et rel.* (37 phylotypes), *F. prausntizii et rel.* (25 phylotypes) and *O. guillermondii et rel.* (20 phylotypes). Bacteroidetes, Actinobacteria, Proteobacteria and Verrucomicrobia were also represented in the core (Figure 5 and Table S5), in line with their established inhabitance in the human gut. Most of the core phylotypes had a highly variable abundance among the healthy individuals (Figure 5C).

### Temporal stability of the common core bacteria

In addition to studying the temporal dynamics of the total microbiota, we defined the temporal variation of the common core phylotypes determined above. We found that the intra-individual CoV of the core phylotypes ranged from 3.3% to 23% indicating higher stability of the core phylotypes compared to all detected phylotypes that were discussed above. The vast majority (70%) of the core phylotypes fluctuated less than 10% during the study (Figure S3). The stable core phylotypes were predominantly members of the Clostridium cluster XIVa but also few taxa belonged to Clostridium cluster IV. The most predominant stable genus-level group included *R. obeum et rel.* and *C. symbiosum et rel.* whose stability was apparent also in the examination based on CoV analysis of the entire microbiota (Table S1). 

We also studied the potential correlations between the abundance and the temporal stability (CoV) of the genus-like taxa. The only group showing strong correlation between the abundance and temporal stability was *F. prauznitzii et rel.* (ρ = −0.66; Figure S4). The negative correlation between the abundance and stability indicated that *F. prauznitzii* was temporally stable only when it was a very abundant member of the microbiota, in contrast to other common gut inhabitants such as Bifidobacteria, which fluctuated in time independently of the abundance (ρ = −0.12; Figure S4). We verified the finding of *F. prauznitzii* with a species-specific qPCR assay, which also indicated strong negative correlation between its abundance and temporal stability (ρ = −0.56).

## Discussion

This study provides the phylogenetic analysis of the intestinal microbiota of subjects with verified intestinal and general health status. The subjects were followed regularly over two months. We utilized a phylogenetic microarray, capable of detection and relative quantification of over thousand intestinal phylotypes [13]. In combination with the health status analysis this allowed us to: 1. Address the composition and temporal dynamics of the GI microbiota, 2. Relate specific microbial groups to the intestinal health status, and 3. Provide new insight into the existence and dimensions of the common core microbiota that we all share.

It has been well established that the human GI microbiota is dominated by Firmicutes and Bacteroidetes followed by Actinobacteria and Verrucomicrobia [8], [12], [35]. The same phyla dominated the GI communities in our study subjects (Results and Figure 2). We detected almost 500 bacterial phylotypes per individual and methanogenic archaea in 80% of the subjects, indicating that our data set should adequately describe the sampled fecal communities based on the current richness estimates [16], [24]. The methanogenic archaea were detected in high prevalence and at variable inter-individual but consistent intra-individual abundance. In contrast to the previous findings [36], our results suggest that the methanogenic archaea are temporally stable members of the GI microbiota and hence may constitute part of the individual core in the carriers. One possible explanation for the mentioned differences is the use of efficient DNA extraction method in this study, resulting into improved detection of the archaea [29].

Our extensive short-term follow-up confirms the low temporal variation of the overall GI microbiota in healthy subjects [10], [11]. High temporal stability suggests a strong selection by the healthy host for the particular assemblage of microbes, additionally supported by the fact that the stable taxa differed across the individuals and hence there was little phylogenetic commonality between the individual cores. However, the stable genus-level taxa may constitute a functionally uniform group as most of them belonged to order Clostridiales, many of which are ubiquitous hydrolytic or fermentative carbohydrate utilizers.

The cluster analysis showed subject-specificity in the bacterial profiles despite the alterations that transiently decreased intra-individual microbiota similarity in few study subjects (Figure 1). Antibiotic treatment is a well-documented modifier of the GI ecosystem [15], [16]. In addition, also the microbial profile collected during non-medicated streptococcal pharyngitis separated clearly from the baseline samples of the affected subject. This finding suggests that infection *per se* may transiently modulate the GI microbiota, supporting a previous, analogous observation associated to fever [37]. As a novel observation, we found that travelling correlated with altered GI microbiota as the similarity of the microbiota profiles decreased significantly following overseas travelling. Various factors could cause the travel-related microbiota instability, including exposure to new environmental microbes, stress or disturbances of the circadian rhythms. Our results in the studied small cohort indicate that normal life changes, such as illness, medication and travelling affect the microbiota homeostasis in a seemingly temporary manner that does not challenge the individuality of the microbiota.

Our results imply that the overall microbiota stability and intestinal symptoms may not be associated to each other. The microbiota profiles of the subjects who reported the most severe intestinal symptoms (Table 1) remained highly stable during the study (Figure 1). Additionally, the instability of the microbiota did not manifest as intestinal symptoms according to the health score records (Table 1), in line with previous studies suggesting that healthy subjects may have an unstable microbiota in the absence of any intestinal complaints [16], [38]. As this study did not involve food diaries, we could not estimate the extent and nature of dietary influences on the temporal stability and the occurrence of the intestinal symptoms. Hence, future studies with dietary records and larger cohorts are needed to verify the findings.

While the occurrence of the intestinal symptoms was not reflected on the stability of the GI microbiota, we identified bacteria whose abundance was statistically significantly associated to the symptoms. The most striking finding at the genus-level was the decreased amount of Bifidobacteria coupled to abdominal pain (Figure 4). Thought the majority of the pain scores indicated only minor disturbance, the reduction in the Bifidobacterial abundance was evident. This study is the first, to our knowledge, to study the correlations between the microbiota composition and intestinal symptoms in healthy individuals, while two studies have analyzed the associations between the IBS patients' GI symptoms and the intestinal bacteria using qPCR [39], [40]. A weak negative association between bifidobacterial counts and the symptoms was described [39], [40] while another study did not find such association [40]. Several studies have reported decreased levels of Bifidobacteria in IBS patients (reviewed in [41]) and a probiotic *B. infantis* is documented to reduce visceral pain both in IBS patients [42] and animal model [43]. The mechanistic background for the ability of Bifidobacteria to eliminate pain is not known but can be speculated in the light of the multiple metabolic traits of Bifidobacteria that impact the host in a beneficial way (reviewed in [44]). Finally, similar to our findings, the IBS studies suggested a role for the Firmicutes in the occurrence of the intestinal symptoms [39], [40]. However, the correlating taxa in the IBS patients (Lactobacilli, *Veillonella* spp. and an uncharacterized *Ruminococcus)* [39], [40] were mostly distinct from ours that were dominated by uncultured and uncharacterized members of *Clostridiaceae, Ruminococcaceae* and *Lachospiraceae.*

This study addressed conceptually as well as concretely the common core microbiota, which may comprise of the taxa that have been selected during the mutual co-evolution of man and his GI microbiota as beneficial partners. Once catalogued, the functional characterization can be focused on these salient microbiota members that possess potential even for therapeutic applications if they are validated to benefit human health and well-being. Our novel approach focusing on the conceptual rather than concrete definition of the common core microbiota show that the core size is highly conditional, depending both on the depth of the analysis and the selected prevalence of the desired phylotypes. The deterministic impact of the coverage of analysis on the common core is supported by the previous finding where doubling the sequencing depth increased the amount of shared species by 25% [8].

By including all the phylotypes above the specified detection limit, we found out that over 40% of the phylotypes were shared among all the nine healthy subjects. The previous sequencing-derived estimates of the share of the shared bacteria have ranged from 0–2% [12], [24] to over 30% [8], [26]. We hypothetize that our large core arises from technical aspects that supported the detection of the shared bacteria as efficient extraction of the community DNA [29] was coupled to its in-depth compositional analysis, covering phylotypes with notably low relative abundance (below 0.05%). Such rare taxa are not accessible with conventional sequencing depth [8], [25] and therefore have often remained undetected. Moreover, our core estimate is arguably less random than the previous ones as it was limited to the previously identified phylotypes due to the microarray technology, and rested upon multiple samples per individual. Hence, in contrast to earlier assumption about the unambiguous dominance of the subject-specific phylotypes in the GI microbiota [12], [45], this study supports a view that a large part of the intestinal bacteria is present in most of the individuals, although in a highly uneven proportions. If so, the individuality of the microbiota would be attributable both to the differential ratios and the pure presence or absence of particular lineages.

In the Finnish subjects analyzed in this study the common core microbiota consisted mainly of uncultured Firmicutes from the Clostridium clusters IV and XIVa, which are also highly represented in the earlier core studies [13], [24], [27]. As a novel observation we found hybridization signal identifying *Oxalibacterium formigenes*, a betaproteobacterium within order Burkholderiales, among the putative core bacteria (Figures 5B and 5C, Table S5). An unclassified Burkholderiales was recently detected as part of the healthy core [26]. *O. formigenes* is one of the few colonic bacteria with well-defined health benefit as it is controls the oxalic acid homeostasis and prevents the formation of kidney stones [46], [47]. Uncharacterized Firmicutes affiliated to the same or related genera that dominated our common core (*Faecalibacterium, Oscillospira*, *Subdoligranulum)* were recently reported as “healthy specific” as their abundance and/or prevalence was discriminative between Crohn's disease patients and healthy controls [48]. In contrast, Bacteroidetes spp. were not an abundant part of the common core microbiota. The previous studies either support [12], [24], [27] or contradict [8], [14], [26] our finding about the low representation of the Bacteroidetes in the common core. While some differences may be attributable to the methodological variation between the studies, our finding of the low proportion of Bacteroidetes (4.5%) in the common core is in line with their high inter- and intra-individual variation [15], [24], [35].

The core phylotypes were temporally more stable than the total pool of the phylotypes, similarly to another recent study [27]. The fact that the stable phylotypes varied among the subjects and only few genus-level taxa were stabile in majority of the healthy subjects (Table S1) support the paradigm of functional redundancy of the GI microbiota, according to which the key processes can be carried out by several taxa. The vast majority of our most stabile genus-level taxa belonged to Clostridial clusters IV and XIVa that contains most of the butyrate producers, testifying for the assured production of this essential metabolite that contributes to the mutualistic homeostasis between the host and the GI microbiota (reviewed in [49]). In our cohort Bifidobacteria were not included in the common core, instead we detected substantial inter- and intra-individual variation that was associated to occurrence of abdominal pain and other intestinal symptoms. Bifidobacteria were in some subjects among the enriched unstable genus-level taxa (Table S2), which is in line with certain previous results [50] but contradicts to many earlier studies, which have reported high temporal stability of this genus [11], [51], [52]. In summary, our findings emphasize the role of Bifidobacteria in the GI ecosystem and warrant their further analysis as regards to the intestinal health. These studies are expected to elucidate also the arisen disparity regarding their intra-individual stability.

Unlike any other genus-level taxon, the temporal stability of the F. prausnitzii group depended on its abundance in the given host. Although *F. prausnitzii et rel.* was abundant in all the individuals (Fig. 5C), it was temporally less stabile in those individuals that had the lowest signal intensity. Our finding may simply reflect situation where *F. prausnitzii et rel.* populations were transiently imbalanced i.e. either declining or re-establishing for a reason or another. *F. prausnitzii* has appeared as a health-associated and colitis-reducing bacterium [2], [48], [53]. Among healthy individuals we did not find evidence for the anticipated negative association between *F. prausnitzii et rel.* and the intestinal symptoms. As the genus *Faecalibacterium* appears functionally more diverse than previously thought [54], its further characterization is required to identify the phylotypes and metabolic features that are essential for the intestinal and general health.

In conclusion, this work extends and defines our current understanding about the normal GI microbiota in healthy subjects by addressing its temporal dynamics, relation to intestinal symptoms and the common core. This knowledge will bring us closer to the definition of intestinal health and the contribution of the GI microbiota within.

## Methods

### Subjects and study design

The study subjects (Table 1) were a subset of healthy Finnish adults (n = 15) from a larger randomized, double-blind, placebo-controlled nine-week intervention trial (Kekkonen et al., unpublished results). Before entering the study, the subjects gave their written informed consent. The study protocol was approved by the Ethics Committee of the Hospital District of Helsinki and Uusimaa (Ethical protocol HUS 357/E0/05). Exclusion criteria were chronic illnesses, gastrointestinal diseases and related medications, use of antibiotics or acute gastrointestinal disorders during the two months before the study, pregnancy, and lactation. The trial consisted of run-in, intervention and follow-up periods, each three week. Fecal samples were collected at six timepoints during seven weeks (at weeks 2,3,4,6,7,9), resulting in a total of 88 samples after two dropouts. During the intervention period the subjects received study drink containing either bacterial strains frequently used in dairy fermentation, namely *Streptococcus thermophilus THS* (n = 4), *Lactococcus lactis ATH74* (n = 2), *Leuconostoc mesenteroides* PIA2 (n = 7), or placebo (n = 2). As the recruitment to this sub-study occurred after randomization in the mother study, we could not affect the number of subjects per intervention group. The subjects were not allowed to consume any probiotics or fermented dairy products during the trial and hence had a restricted exposure to the dietary microbes.

Before the data analysis, we tested the possible effect of the intervention on the microbiota. No statistically significant difference between the intervention groups were observed with the analysis of variance (ANOVA) that was applied to the phylotype-level data above the specified threshold (log10 value of 1.8; see below). However, when also the phylotypes below the detection threshold were included, a single statistically significant (q<0.005) difference was observed: The Leuconostoc-like phylotypes transiently increased during the intervention in the group consuming L. mesenteroides PIA2. While the result testified for the high resolution of the HITChip analysis, we only addressed the signals above the background in the present analysis, and thus the subjects were considered to constitute a uniform group in terms of the microbiota composition.

At the time of each fecal sampling the subjects filled a Health-Related Quality of Life (HRQoL) -questionnaire, which was based on the RAND-36 questionnaire [55] and was supplemented with questions assessing the GI symptoms as adapted from the Irritable Bowel Syndrome Questionnaire [56]. The questions covered general and emotional health with focus on the intestinal symptoms whose occurrence and impact on daily activities were assessed with 14 questions (listed in Figure 3) using 5-step Likert scale (e.g. 1 =  no symptom, 5 =  very severe disturbance). The recall period was one or two weeks according to the sampling intervals. The health scores were obtained by scoring each question from 0 to 100 and then averaging related questions to form the general, intestinal and emotional health score. In addition, the participants provided data that covered possible illness, medication and travelling abroad during the study.

### Fecal samples and DNA extraction

The fecal samples we collected at home, immediately frozen at −20°C, and transported within 12 hours to the study center where they were stored at −70°C until analyses. Community DNA was extracted from the fecal samples with a repeated bead beating method that has been recently validated [29]. The extracted DNA was quantified with a Nanodrop spectrophotometer (NanoDrop Technologies, Wilmington, DE, USA) and analyzed by 0.8% agarose gel electrophoresis for integrity.

### Microarray analysis

The HITChip microarray analyses were performed as previously described [13]. In brief, the HITChip consists of over 4 800 oligonucleotide probes targeting the V1 and V6 hypervariable regions of the 16S rRNA gene, spotted in duplo on the custom Agilent arrays (Agilent Technologies, Palo Alto, CA, USA). To prepare the fecal DNAs for the microarray analysis, full-length 16S rRNA gene was amplified using primers T7prom-Bact-27-for and Uni-1492-rev [13]. The PCR products were transcribed into RNA and labeled with Cy3 and Cy5 prior to fragmentation and hybridization on the array. The arrays were scanned with Agilent DNA Microarray Scanner and the data were extracted from the generated images using the Agilent Extraction Software 9.1.

### Computational pre-processing of the microarrays

The array normalization was conducted as described earlier [13], [29] including spatial normalization with polynomial regression of the each scanner channel followed by outlier detection and quantile normalization of both of the Cy3 and Cy5 dyes for each sample. The duplicates that showed over 0.98 Pearson correlations between the dyes were considered for further analysis and the dyes were averaged, otherwise the hybridization was reproduced. The between-array normalization of the samples was conducted by using the assumption of normal-exponential (background-signal) distribution and quantile normalization [57]. The array probes were organized based on their 16S rRNA sequences on three levels of phylogeny as described before [13]. In brief, the HITChip signal intensity was analyzed using the following phylogenetic assignment levels: 1) the phylum-level, with the specification of Firmicutes down to Clostridium clusters, creating altogether 23 groups; 2) the genus-like level, including 131 groups of sequences with ≥90% sequence identity, and 3) the phylotype (species-like) level with 1033 distinct phylotypes with ≥98% sequence similarity to cultured species or clones corresponding to uncultured micro-organisms. “Genus-level taxa with ≥90% sequence identity distributed over multiple genera are termed “*et rel*. ”. To reduce the experimental and potential cross-hybridization noise, and to reliably assess whether certain phylotypes were present in a sample based on the hybridization intensity distributions of all the subjects, we estimated the signal intensity threshold from the data using the approach originally developed for gene expression microarrays [58]. The thus obtained signal intensity threshold was log10 intensity of 1.8 (Figure S1), corresponding to estimated 0.02% of the total signal as a detection limit for each phylotype. To increase the analysis reliability, we additionally determined the probe specificities for each phylotype by calculating the number of the targeted phylotypes per probe in order to specify taxa that are potentially subject to cross-hybridization (i.e. are detected with probes that have more than one target phylotype based on the hypervariable V1 and V6 sequences of the 16S rDNA). Multiple targeted phylotypes were essentially detected only within the same genus-level taxa. Additionally, the Robust Probabilistic Averaging algorithm was used to estimate the reliability of the individual HITChip probes in regard to noise attributable e.g. to unintended cross-hybridization with non-target sequences [34], potentially resulting in false positives in the common core analysis. Previously, a good correlation between the HITChip- and pyrosequencing-derived microbial profiles has been recorded [30], [59].

### Quantitative PCR analysis

Triplicate quantitative PCR (qPCR) amplifications of the 16S rRNA gene were performed for all available samples using the MX3005P Real-Time PCR System (Stratagene, La Jolla, CA, USA) and CYBR green chemistry. Total bacteria were quantified by amplifying 0.5 ng of fecal DNA with universal primers [60]. The archaea were quantified using primers specific for the genus *Methanobrevibacter* [61] and 25 ng of the template DNA. The assays for *Faecalibacterium prausnitzii* and *Bifidobacterium* spp. were conducted as described previously [62]. The reaction conditions and construction of standard curves have been previously described [29], [62]. The data are expressed as 16S rRNA gene copies µg-1 template DNA.

### Data analyses

The data analyses were performed in R version 2.10.1 [63] and Microsoft Excel. The statistical analysis methods used in this study included linear models with ANOVA (for intervention effects) and t-tests [64], [65] for normally distributed data, Wilcoxon [66] test for non-normal data, determination of false discovery rate of p-values as q-values [67] and statistical over-representation (enrichment) of genus-level groups in phylotypes-level lists using Fisher's exact test [68]. We visualized the data by using hierarchical clustering and heatmap analyses. The hierarchical clustering was used to group the samples according to the correlation distance and average clustering criterion.

All data analyses were conducted with log-transformed data, with exception of the estimation of the percentual abundances from the total signal and calculation of the signal fold changes in relation to bloating and abdominal pain. We determined the microbial stability by calculating the intra- and inter-individual Pearson correlations (r), and the temporal variation of the individual phylotypes by calculating the Coefficient of Variation (CoV) within a subject from all timepoints. Mean standard deviation was calculated for the correlations and expressed in the text as ±.

To analyze the significant correlations between the microbial profiles and the intestinal symptoms in the HRQoL-questionnaire we calculated their Spearman's rank correlation coefficient (ρ). All HRQoL items are scored so that a high score indicates a good health status (22), which resulted in non-intuitive direction of correlations i.e. in negative correlation upon simultaneous increase of the bacterial abundance and symptom severity due to the drop of the HRQoL score. Hence, to truthfully express the association between the symptoms and the microbiota data, the direction of resultant correlations (ρ) was inversed from negative to positive and vice versa. Using the HRQoL GI symptom scores, we divided the samples into two groups: with and without concurrent abdominal pain and bloating, and then used these groups to analyse the association between microbiota composition and the two most commonly recorded intestinal symptoms. We analyzed the common core microbiota with the phylotype-level data using adjustable parameters for the abundance (relative contribution to the total signal) and prevalence (number of affected subjects). The resulting surface was visualized with a perspective plot [69]. For all analyses, p-values below 0.05 and q-values below 0.005 were considered significant.

## Supporting Information

Figure S1 **HITChip signal intensity distribution of all phylotypes in the entire data set (15 subjects, 88 samples).** The red line indicates the threshold of log_10_ intensity >1.8, above which any phylotype was considered to be present. Altogether 687 phylotypes passed the treshold, representing 66.5% of the phylotypes detectable with the HITChip.
(TIF)

Figure S2 **Effect of travelling on the microbiota stability.** Similarity of the HITChip fingerprints between two consecutive timepoints of each traveller is expressed using Pearson correlation.
(TIF)

Figure S3 **Temporal variation of the core phylotypes.** The line indicates Coefficient of Variation (CoV) of the common core phylotypes. The phylotypes with less than 10% variation within the seven week study period are summed up to phylum level.
(TIF)

Figure S4 **Correlation between temporal stability and abundance.** Vertical lines indicate absence of correlation between the temporal stability (CoV) and abundance (%) for the genus-level taxa specified in the box. In the case of *F. Prausnitzii* group, the line connects high values on both axes and visualizes the negative correlation between the two parameters.
(TIF)

Table S1 **Statistically enriched stable genus-like taxa from nine healthy subjects.**
(DOCX)

Table S2 **Statistically enriched unstable genus-like taxa from nine healthy subjects.**
(DOCX)

Table S3 **List of phylotypes correlating significantly with bloating.**
(DOCX)

Table S4 **List of phylotypes correlating significantly with abdominal pain.**
(DOCX)

Table S5 **Phylotypes in the common core microbiota; shared between nine healthy subjects and contributing over 0.03% to the total signal intensity.**
(DOCX)
