# Supplementary figures and images for: Intestinal Microbiota in Healthy Adults: Temporal Analysis Reveals Individual and Common Core and Relation to Intestinal Symptoms
Source: PLoS One. 2011 Jul 28;6(7):e23035. doi: 10.1371/journal.pone.0023035 (PMC3145776; doi:10.1371/journal.pone.0023035)

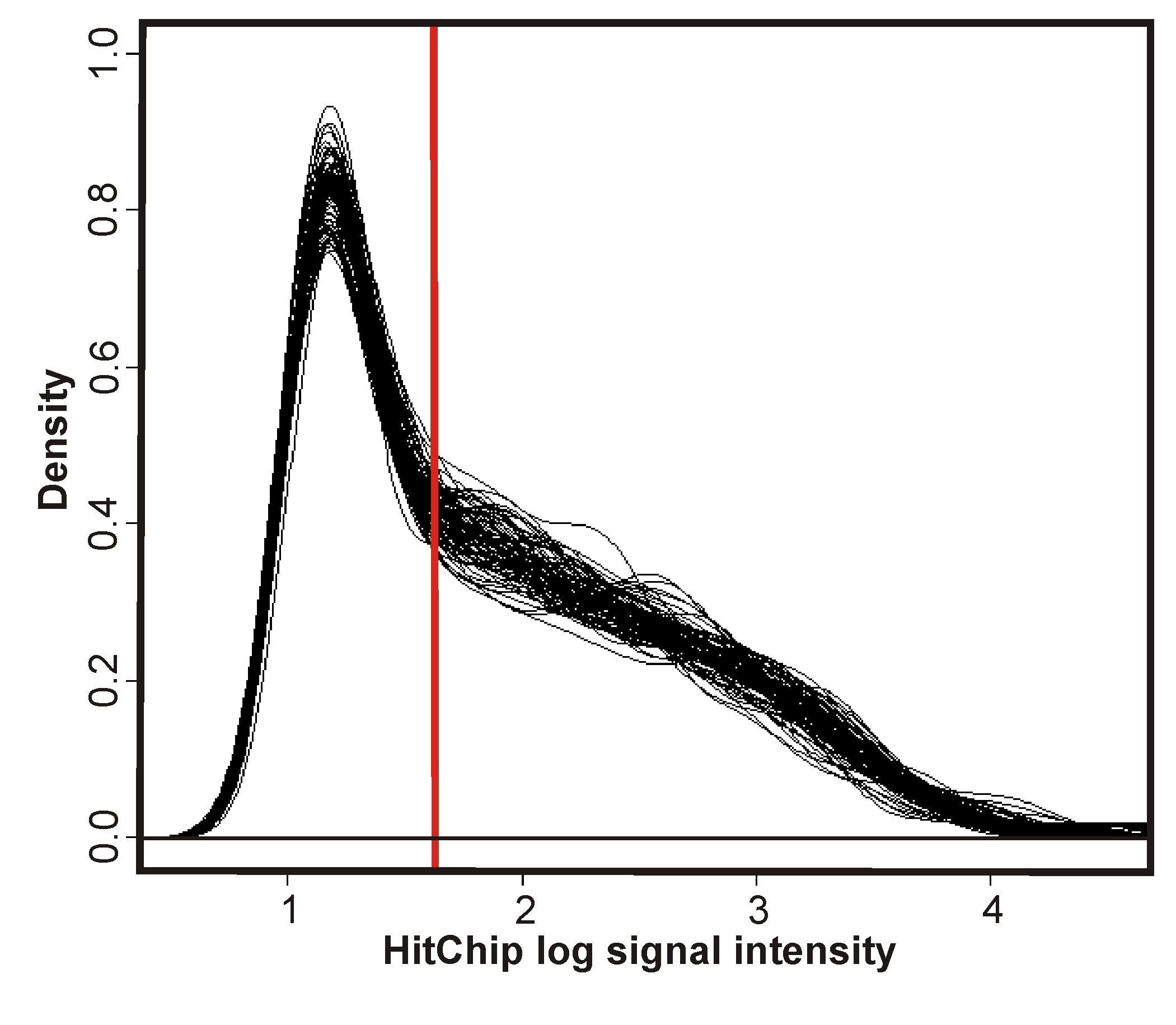

Supplement: Figure S1 — HITChip signal intensity distribution of all phylotypes in the entire data set (15 subjects, 88 samples). The red line indicates the threshold of log10 intensity >1.8, above which any phylotype was considered to be present. Altogether 687 phylotypes passed the treshold, representing 66.5% of the phylotypes detectable with the HITChip. (TIF) [file pone.0023035.s001.tif]

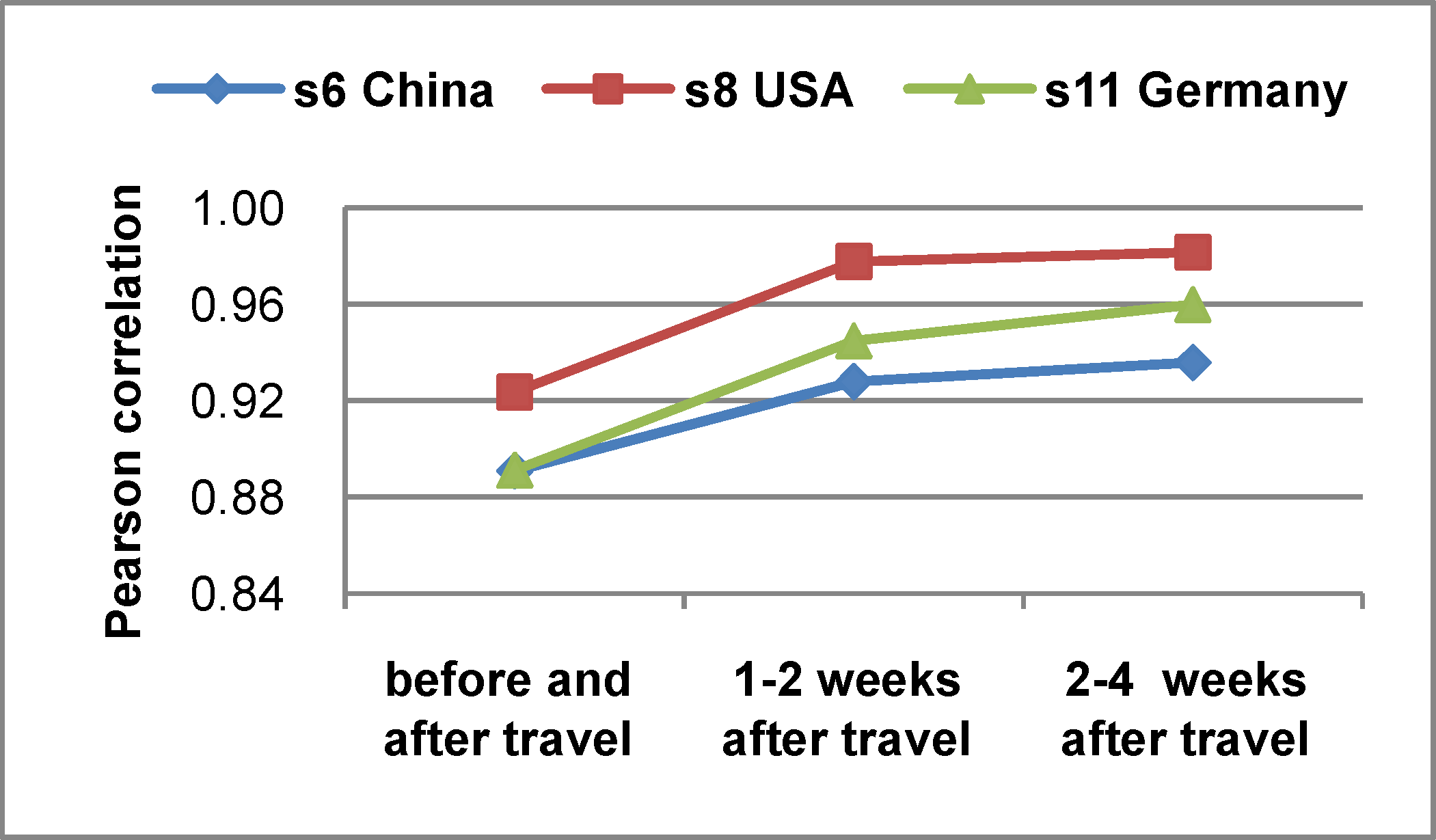

Supplement: Figure S2 — Effect of travelling on the microbiota stability. Similarity of the HITChip fingerprints between two consecutive timepoints of each traveller is expressed using Pearson correlation. (TIF) [file pone.0023035.s002.tif]

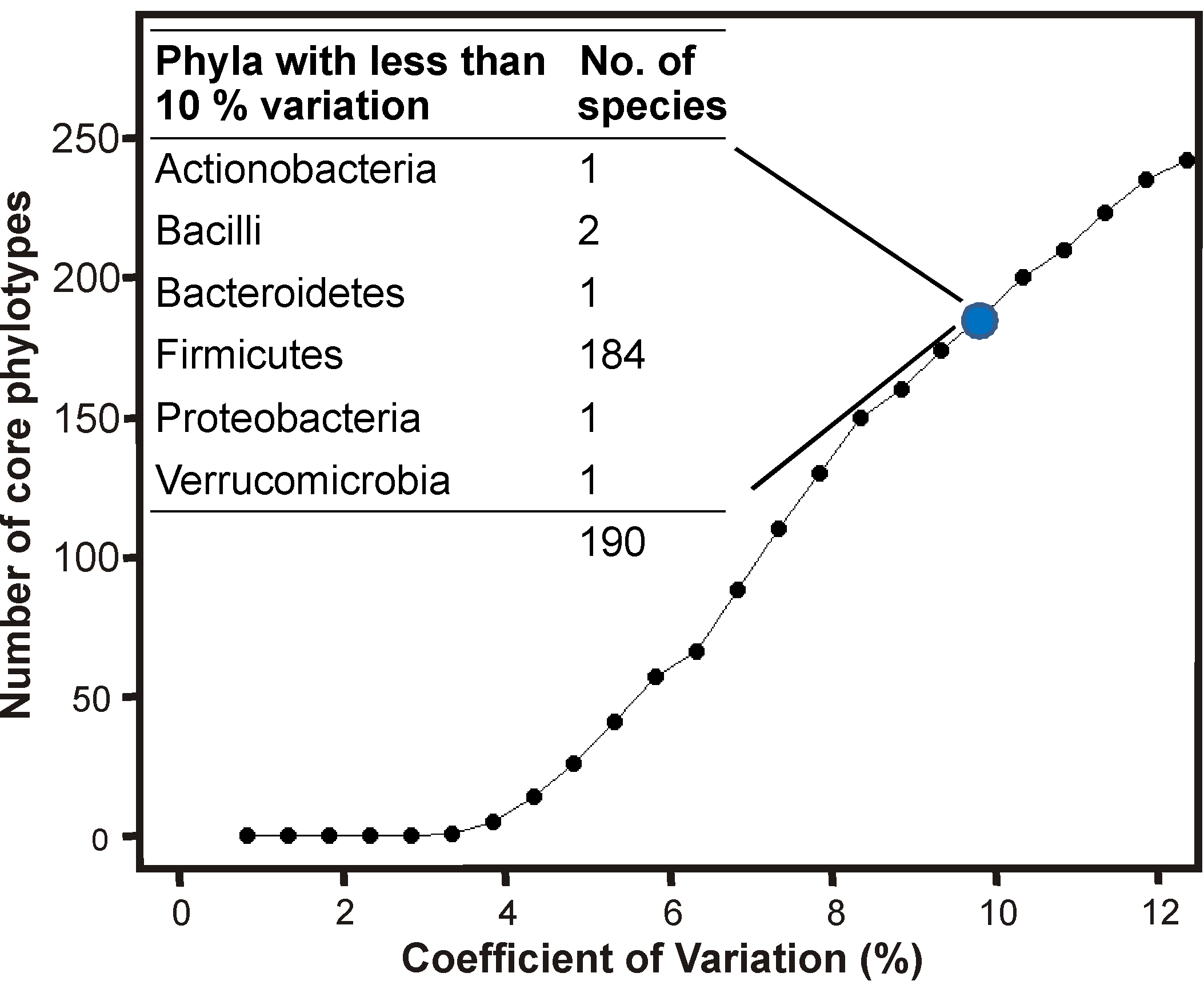

Supplement: Figure S3 — Temporal variation of the core phylotypes. The line indicates Coefficient of Variation (CoV) of the common core phylotypes. The phylotypes with less than 10% variation within the seven week study period are summed up to phylum level. (TIF) [file pone.0023035.s003.tif]

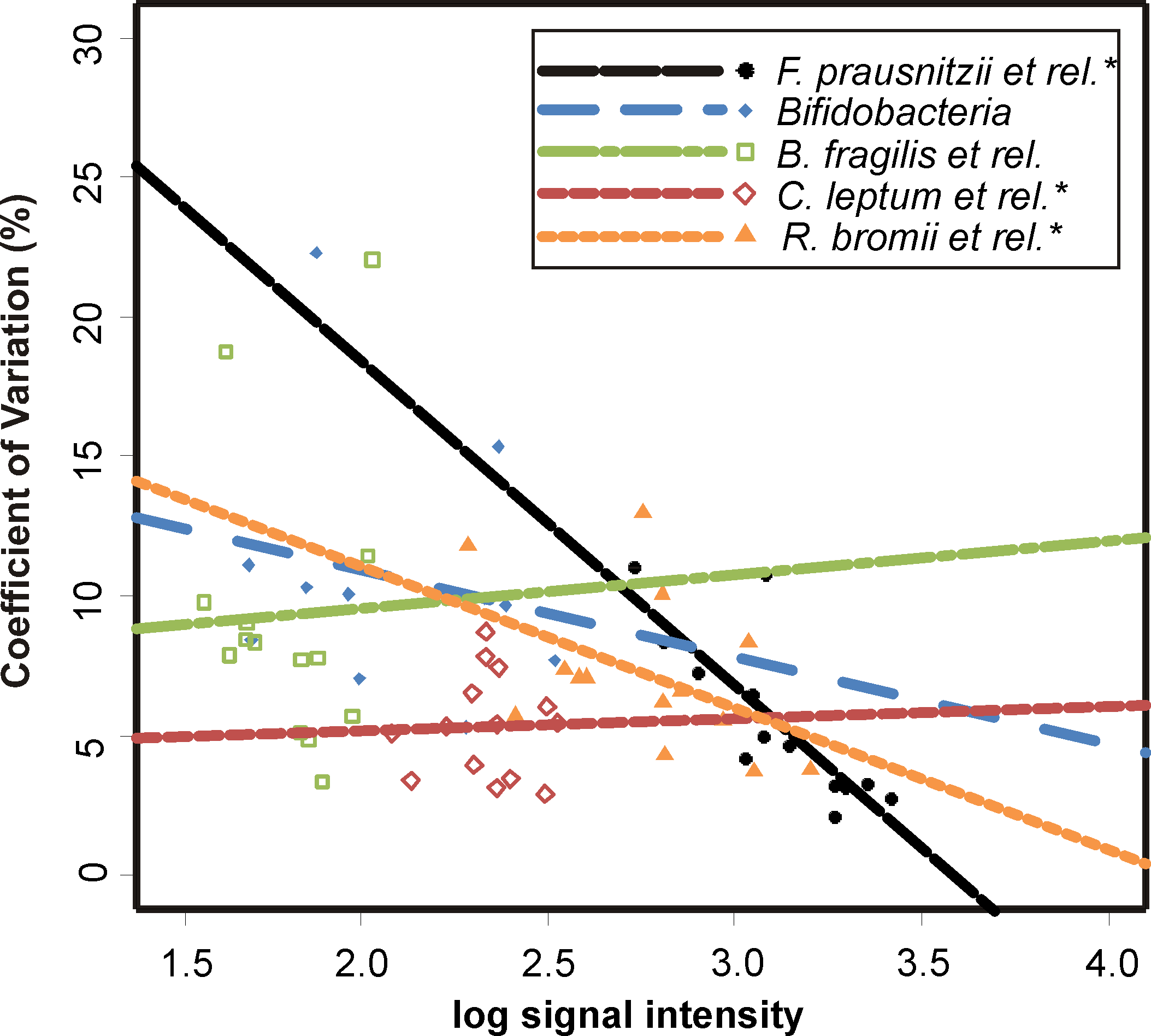

Supplement: Figure S4 — Correlation between temporal stability and abundance. Vertical lines indicate absence of correlation between the temporal stability (CoV) and abundance (%) for the genus-level taxa specified in the box. In the case of F. Prausnitzii group, the line connects high values on both axes and visualizes the negative correlation between the two parameters. (TIF) [file pone.0023035.s004.tif]
