# Supplementary material for: Intestinal Microbiota in Healthy Adults: Temporal Analysis Reveals Individual and Common Core and Relation to Intestinal Symptoms
Source: PLoS One. 2011 Jul 28;6(7):e23035. doi: 10.1371/journal.pone.0023035 (PMC3145776; doi:10.1371/journal.pone.0023035)
Supplement: Table S1 — Statistically enriched stable genus-like taxa from nine healthy subjects. (DOCX) [file pone.0023035.s005.docx]

**Table S1.** Statistically enriched stable genus-like taxa from nine healthy subjects

|  |  | **CoV (%)** | | |
| --- | --- | --- | --- | --- |
|  | **No. of subjects** | **Average** | **Min** | **Max** |
| *Eubacterium hallii et rel.** | 1 | 4.6 | 3.0 | 7.7 |
| *Oscillospira guillermondii et rel.* | 1 | 6.0 | 4.3 | 8.2 |
| *Papillibacter cinnamivorans et rel.* | 1 | 6.4 | 2.7 | 12.7 |
| *Roseburia intestinalis et rel.** | 1 | 6.6 | 3.9 | 9.2 |
| *Clostridium cellulosi et rel.** | 2 | 5.7 | 3.0 | 7.7 |
| *Clostridium nexile et rel.* | 2 | 4.3 | 3.5 | 5.5 |
| *Clostridium sphenoides et rel.* | 2 | 5.1 | 3.6 | 7.8 |
| *Eubacterium ventriosum et rel.* | 2 | 5.8 | 3.0 | 10.5 |
| *Lachnospira pectinoschiza et rel.* | 2 | 5.9 | 3.4 | 8.5 |
| *Streptococcus mitis et rel.** | 2 | 5.9 | 2.8 | 11.7 |
| *Subdoligranulum variable at rel.** | 2 | 5.9 | 3.1 | 8.5 |
| *Dorea formicigenerans et rel.** | 3 | 4.5 | 3.3 | 6.2 |
| *Faecalibacterium prausnitzii et rel.** | 3 | 5.8 | 2.1 | 11.0 |
| *Clostridium leptum et rel.** | 4 | 4.7 | 2.9 | 7.8 |
| *Coprococcus eutactus et rel.** | 4 | 4.3 | 2.9 | 5.9 |
| *Streptococcus bovis et rel.* | 4 | 6.7 | 3.3 | 17.9 |
| *Clostridium symbiosum et rel.** | 7 | 4.4 | 3.5 | 6.1 |
| *Ruminococcus obeum et rel.** | 7 | 3.7 | 2.5 | 5.2 |

*includes signal from probes that are not phylotype-specific (see Methods)
