# Supplementary material for: Intestinal Microbiota in Healthy Adults: Temporal Analysis Reveals Individual and Common Core and Relation to Intestinal Symptoms
Source: PLoS One. 2011 Jul 28;6(7):e23035. doi: 10.1371/journal.pone.0023035 (PMC3145776; doi:10.1371/journal.pone.0023035)
Supplement: Table S2 — Statistically enriched unstable genus-like taxa from nine healthy subjects. (DOCX) [file pone.0023035.s006.docx]

**Table S2.** Statistically enriched unstable genus-like taxa from nine healthy subjects

|  |  | **CoV (%)** | | |
| --- | --- | --- | --- | --- |
|  | **No. of subjects** | **Average** | **Min** | **Max** |
| *Anaerotruncus colihominis et rel.* | 1 | 6.46 | 3.90 | 9.51 |
| *Bacteroides fragilis et rel.* | 1 | 8.52 | 3.32 | 22.04 |
| *Bifidobacterium* | 1 | 9.33 | 5.30 | 15.36 |
| *Bryantella formatexigens et rel.* | 1 | 5.74 | 4.41 | 8.67 |
| *Butyrivibrio crossotus et rel.* | 1 | 7.34 | 4.26 | 10.93 |
| *Eubacterium rectale et rel.* | 1 | 5.64 | 3.66 | 7.37 |
| *Papillibacter cinnamivorans et rel.* | 1 | 6.40 | 2.68 | 12.65 |
| *Parabacteroides distasonis et rel.* | 1 | 7.18 | 4.66 | 12.21 |
| *Prevotella melaninogenica et rel.* | 1 | 8.17 | 5.56 | 15.48 |
| *Roseburia intestinalis et rel.** | 1 | 6.57 | 3.95 | 9.22 |
| *Veillonella* | 1 | 16.34 | 16.34 | 16.34 |
| *Bacteroides intestinalis et rel.* | 2 | 8.08 | 4.96 | 16.03 |
| *Bacteroides ovatus et rel.* | 2 | 8.72 | 3.10 | 20.50 |
| *Clostridia* | 2 | 15.30 | 11.77 | 17.87 |
| *Eubacterium siraeum et rel.* | 2 | 13.01 | 5.91 | 25.30 |
| *Eubacterium ventriosum et rel.* | 2 | 5.79 | 2.97 | 10.48 |
| *Faecalibacterium prausnitzii et rel.** | 2 | 5.81 | 2.11 | 11.05 |
| *Lachnobacillus bovis et rel.** | 2 | 7.40 | 5.32 | 10.60 |
| *Sporobacter termitidis et rel.** | 2 | 6.58 | 4.92 | 9.16 |
| *Streptococcus bovis et rel.* | 2 | 6.72 | 3.29 | 17.91 |
| *Subdoligranulum variable at rel.** | 2 | 5.95 | 3.08 | 8.54 |
| *Allistipes et rel.* | 3 | 7.33 | 4.14 | 11.58 |
| *Lachnospira pectinoschiza et rel.** | 3 | 5.87 | 3.43 | 8.46 |
| *Bacteroides vulgatus et rel.* | 4 | 9.90 | 4.08 | 20.12 |
| *uncultured Clostridiales I* | 4 | 17.30 | 8.52 | 31.20 |
| *uncultured Clostridiales II* | 4 | 9.18 | 5.23 | 14.07 |

*includes signal from probes that are not phylotype-specific (see Methods)
