# Supplementary material for: Intestinal Microbiota in Healthy Adults: Temporal Analysis Reveals Individual and Common Core and Relation to Intestinal Symptoms
Source: PLoS One. 2011 Jul 28;6(7):e23035. doi: 10.1371/journal.pone.0023035 (PMC3145776; doi:10.1371/journal.pone.0023035)
Supplement: Table S3 — List of phylotypes correlating significantly with bloating. (DOCX) [file pone.0023035.s007.docx]

**Table S3.** List of phylotypes correlating significantly with bloating.

| **Phylum/**  **Order** | **Genus-like group** | **Phylotype** | **Intensity if bloating** | **Intensity if no bloating** | **Fold change** | **Correlation** | **q-value** |
| --- | --- | --- | --- | --- | --- | --- | --- |
| Clostridium cluster IV | *Anaerotruncus colihominis et rel.* | *bacterium adhufec101* | 17817 | 1592 | 11.2 | 0.51 | 9.10E-05 |
|  |  | *Uncultured bacterium clone Eldhufec215* | 31233 | 2257 | 13.8 | 0.57 | 8.40E-06 |
|  | *Oscillospira guillermondii et rel.* | *uncultured bacterium D288* | 90105 | 36704 | 2.5 | 0.41 | 2.60E-03 |
|  |  | *uncultured bacterium HuRC81* | 90105 | 36704 | 2.5 | 0.41 | 2.60E-03 |
|  |  | *uncultured bacterium D440* | 1357 | 585 | 2.3 | 0.45 | 6.20E-04 |
|  |  | *uncultured bacterium MA30* | 334089 | 99149 | 3.4 | 0.45 | 5.80E-04 |
|  |  | *uncultured bacterium OLDA-H2* | 418623 | 91557 | 4.6 | 0.46 | 3.90E-04 |
|  |  | *uncultured bacterium OLDB-B7* | 418623 | 91557 | 4.6 | 0.46 | 3.90E-04 |
|  | *Ruminococcus callidus et rel.* | *Ruminococcus flavefaciens* | 125904 | 10155 | 12.4 | 0.54 | 4.00E-05 |
|  |  | *uncultured bacterium D789* | 1431 | 146 | 9.8 | 0.58 | 3.70E-06 |
|  | *Sporobacter termitidis et rel.* | *Uncultured bacterium UC7-1* | 59266 | 8373 | 7.1 | 0.42 | 1.80E-03 |
|  |  | *uncultured bacterium HuCB5* | 32080 | 112512 | 3.5 | -0.51 | 9.10E-05 |
|  | *Subdoligranulum variable at rel.* | *uncultured Gram-positive bacterium NB4C12* | 12233 | 70660 | 5.8 | -0.42 | 1.50E-03 |
|  |  |  |  |  |  |  |  |
|  |  |  |  |  |  |  |  |
|  | *Butyrivibrio crossotus et rel.* | *Uncultured bacterium clone Eldhufec138* | 7639 | 1112 | 6.9 | 0.4 | 3.50E-03 |
|  |  | *uncultured bacterium HuCB40* | 3121 | 527 | 5.9 | 0.44 | 7.90E-04 |
| Clostridium cluster XIVa | *Clostridium sphenoides et rel.* | *uncultured bacterium HuCA27* | 16062 | 32937 | 2.1 | -0.43 | 1.20E-03 |
|  |  | *uncultured bacterium OLDA-H6* | 2010 | 600 | 3.4 | 0.39 | 3.70E-03 |
|  |  | *uncultured bacterium HuDI16* | 14899 | 4289 | 3.5 | 0.5 | 1.30E-04 |
|  | *Clostridium symbiosum et rel.* | *uncultured bacterium B840* | 9996 | 4105 | 2.4 | 0.44 | 7.30E-04 |
|  |  | *uncultured bacterium B147* | 580 | 189 | 3.1 | 0.52 | 6.80E-05 |
|  |  | *uncultured Gram-positive bacterium NO59* | 36323 | 5156 | 7.0 | 0.63 | 1.80E-07 |
|  |  | *Clostridium symbiosum* | 42914 | 6747 | 6.4 | 0.63 | 1.80E-07 |
|  | *Eubacterium rectale et rel.* | *uncultured bacterium M372* | 456 | 974 | 2.1 | -0.41 | 2.10E-03 |
|  | *Lachnospira pectinoschiza et rel.* | *uncultured bacterium Adhufec019rbh* | 5358 | 360 | 14.9 | 0.41 | 2.50E-03 |
|  | *Ruminococcus lactaris et rel.* | *Ruminococcus lactaris* | 1815 | 5925 | 3.3 | -0.44 | 9.50E-04 |
