# Supplementary material for: Intestinal Microbiota in Healthy Adults: Temporal Analysis Reveals Individual and Common Core and Relation to Intestinal Symptoms
Source: PLoS One. 2011 Jul 28;6(7):e23035. doi: 10.1371/journal.pone.0023035 (PMC3145776; doi:10.1371/journal.pone.0023035)
Supplement: Table S4 — List of phylotypes correlating significantly with abdominal pain. (DOCX) [file pone.0023035.s008.docx]

|  | *Ruminococcus lactaris et rel.* | *Ruminococcus lactaris* | 1814.87 | 5925.1 | 3.26 | -0.44 | 9.50E-04 |
| --- | --- | --- | --- | --- | --- | --- | --- |

**Table S4.** List of phylotypes correlating significantly with abdominal pain.

| **Phylum/Order** | **Genus-like group** | **Phylotype** | **Intensity if pain** | **Intensity if no pain** | **Fold change** | **Correlation** | **q-value** |
| --- | --- | --- | --- | --- | --- | --- | --- |
| Actinobacteria | *Bifidobacterium* | *Uncultured bacterium clone Eldhufec085* | 2233 | 17476 | 7.8 | -0.44 | 8.90E-04 |
|  |  | *uncultured Bifidobacterium sp. 16B* | 2233 | 17476 | 7.8 | -0.44 | 8.90E-04 |
|  |  | *Bifidobacterium thermophilum* | 700 | 5200 | 7.4 | -0.41 | 2.50E-03 |
|  |  | *uncultured Bifidobacterium sp. 15D* | 514 | 3405 | 6.6 | -0.48 | 2.50E-04 |
|  |  | *Bifidobacterium adolescentis* | 210 | 1321 | 6.3 | -0.42 | 1.70E-03 |
|  |  | *uncultured bacterium Adhufec069rbh* | 307 | 1838 | 6.0 | -0.48 | 2.50E-04 |
|  |  | *uncultured bacterium (human infant) N14A* | 95 | 556 | 5.9 | -0.45 | 6.50E-04 |
|  |  | *uncultured Bifidobacterium sp. 9A* | 471 | 2541 | 5.4 | -0.5 | 1.40E-04 |
|  |  | *Bifidobacterium catenulatum* | 158 | 794 | 5.0 | -0.43 | 1.10E-03 |
|  |  | *Bifidobacterium dentium* | 166 | 782 | 4.7 | -0.49 | 1.70E-04 |
|  |  | *Uncultured bacterium clone Eldhufec088* | 125 | 561 | 4.5 | -0.44 | 7.70E-04 |
|  |  | *uncultured Bifidobacterium sp. 13D* | 125 | 561 | 4.5 | -0.44 | 7.70E-04 |
|  |  | *uncultured Bifidobacterium sp. 16C* | 125 | 561 | 4.5 | -0.44 | 7.70E-04 |
|  |  | *uncultured Bifidobacterium sp. 9C* | 125 | 561 | 4.5 | -0.44 | 7.70E-04 |
|  |  |  |  |  |  |  |  |
| Bacteroidetes | *Tannerella et rel.* | *uncultured bacterium NI77* | 830 | 141 | 5.9 | 0.42 | 1.60E-03 |
|  |  |  |  |  |  |  |  |
|  |  |  |  |  |  |  |  |
| Clostridium cluster IV | *Anaerotruncus colihominis et rel.* | *Uncultured bacterium clone Eldhufec215* | 31233 | 2257 | 13.8 | 0.59 | 3.40E-06 |
|  |  | *bacterium adhufec101* | 17817 | 1592 | 11.2 | 0.51 | 7.60E-05 |
|  |  |  |  |  |  |  |  |
|  | *Faecalibacterium prausnitzii et rel.* | *uncultured bacterium KP66* | 139708 | 48234 | 2.9 | 0.4 | 3.50E-03 |
|  |  | *uncultured bacterium L420* | 12161 | 4955 | 2.5 | 0.4 | 3.00E-03 |
|  | *Oscillospira guillermondii et rel.* | *uncultured bacterium MA30* | 334089 | 99149 | 3.4 | 0.41 | 2.40E-03 |
|  |  |  |  |  |  |  |  |
|  | *Ruminococcus callidus et rel.* | *Ruminococcus flavefaciens* | 125904 | 10155 | 12.4 | 0.52 | 7.20E-05 |
|  |  | *uncultured bacterium D789* | 1431 | 146 | 9.8 | 0.46 | 4.10E-04 |
|  |  |  |  |  |  |  |  |
|  | *Subdoligranulum variable at rel.* | *uncultured bacterium HuCB5* | 32080 | 112512 | 3.5 | -0.42 | 1.60E-03 |
|  |  |  |  |  |  |  |  |
| Clostridium cluster XIVa | *Clostridium sphenoides et rel.* | *uncultured bacterium HuDI16* | 14899 | 4289 | 3.5 | 0.4 | 3.40E-03 |
|  |  | *uncultured bacterium HuCA27* | 16062 | 32937 | 2.1 | -0.44 | 1.00E-03 |
|  | *Clostridium symbiosum et rel.* | *uncultured Gram-positive bacterium NO59* | 36323 | 5156 | 7.0 | 0.54 | 3.70E-05 |
|  |  | *Clostridium symbiosum* | 42914 | 6747 | 6.4 | 0.57 | 8.40E-06 |
|  | *Dorea formicigenerans et rel.* | *uncultured bacterium N874* | 2714 | 1642 | 1.7 | 0.4 | 3.20E-03 |
|  |  |  |  |  |  |  |  |
|  | *Ruminococcus lactaris et rel.* | *Ruminococcus sp. CO41* | 1976 | 22434 | 11.4 | -0.41 | 2.70E-03 |
|  |  |  |  |  |  |  |  |
| Uncultured Clostridiales | *Uncultured Clostridiales II* | uncultured bacterium OLDB-H1 | 3160 | 122 | 25.9 | 0.53 | 5.00E-05 |
