# Supplementary material for: Intestinal Microbiota in Healthy Adults: Temporal Analysis Reveals Individual and Common Core and Relation to Intestinal Symptoms
Source: PLoS One. 2011 Jul 28;6(7):e23035. doi: 10.1371/journal.pone.0023035 (PMC3145776; doi:10.1371/journal.pone.0023035)
Supplement: Table S5 — Phylotypes in the common core microbiota; shared between nine healthy subjects and contributing over 0.03% to the total signal intensity. (DOCX) [file pone.0023035.s009.docx]

**Table S5.** Phylotypes in the common core microbiota; shared between nine healthy subjects and contributing over 0.03% to the total signal intensity.

| **Phylum/Order** | **Genus-like group** | **Number of phylotypes** |
| --- | --- | --- |
| **Actinobacteria** |  | **2** |
|  | *Collinsella* | 2 |
| **Bacilli** |  | **15** |
|  | *Streptococcus bovis et rel.* | 9 |
|  | *Streptococcus intermedius et rel.* | 1 |
|  | *Streptococcus mitis et rel.** | 5 |
| **Bacteroidetes** |  | **13** |
|  | *Allistipes et rel.* | 2 |
|  | *Bacteroides plebeius et rel.* | 1 |
|  | *Bacteroides uniformis et rel.* | 1 |
|  | *Bacteroides vulgatus et rel.* | 8 |
|  | *Parabacteroides distasonis et rel.* | 1 |
| **Clostridium cluster IV** |  | **111** |
|  | *Anaerotruncus colihominis et rel.* | 2 |
|  | *Clostridium cellulosi et rel.** | 8 |
|  | *Clostridium leptum et rel.** | 8 |
|  | *Clostridium orbiscindens et rel.* | 11 |
|  | *Faecalibacterium prausnitzii et rel.** | 25 |
|  | *Oscillospira guillermondii et rel.* | 20 |
|  | *Papillibacter cinnamivorans et rel.* | 4 |
|  | *Ruminococcus bromii et rel.** | 3 |
|  | *Ruminococcus callidus et rel.** | 3 |
|  | *Sporobacter termitidis et rel.** | 13 |
|  | *Subdoligranulum variable at rel.** | 14 |
| **Clostridium cluster XIVa** |  | **145** |
|  | *Anaerostipes caccae et rel.* | 5 |
|  | *Bryantella formatexigens et rel.* | 5 |
|  | *Butyrivibrio crossotus et rel.* | 4 |
|  | *Clostridium colinum et rel.* | 1 |
|  | *Clostridium nexile et rel.* | 7 |
|  | *Clostridium sphenoides et rel.* | 8 |
|  | *Clostridium symbiosum et rel.** | 16 |
|  | *Coprococcus eutactus et rel.** | 9 |
|  | *Dorea formicigenerans et rel.** | 12 |
|  | *Eubacterium hallii et rel.** | 4 |
|  | *Eubacterium rectale et rel.* | 6 |
|  | *Eubacterium ventriosum et rel.* | 3 |
|  | *Lachnobacillus bovis et rel.** | 7 |
|  | *Lachnospira pectinoschiza et rel.** | 9 |
|  | *Outgrouping clostridium cluster XIVa* | 4 |
|  | *Roseburia intestinalis et rel.** | 4 |
|  | *Ruminococcus gnavus et rel.* | 2 |
|  | *Ruminococcus lactaris et rel.* | 2 |
|  | *Ruminococcus obeum et rel.** | 37 |
| **Proteobacteria** |  | **1** |
|  | *Oxalobacter formigenes et rel.** | 1 |
| **Verrucomicrobia** |  | **1** |
|  | *Akkermansia* | 1 |

*includes signal from probes that are not phylotype-specific (see Methods)
